# Pharmacognostic evaluation and antimicrobial activity of *Pteridium aquilinum* (L.) Kuhn leaves (*Onocleaceae*) via *in vitro* and *in silico* perspectives

**DOI:** 10.1371/journal.pone.0318943

**Published:** 2025-04-09

**Authors:** Oluwatoyin Temilolu Adebayo, Bolaji Bosede Oluremi, Akingbolabo Daniel Ogunlakin, Gideon Ampoma Gyebi, Mubo Adeola Sonibare

**Affiliations:** 1 Department of Pharmacognosy, Faculty of Pharmacy, University of Ibadan, Ibadan, Nigeria; 2 Department of Pharmaceutical Microbiology, Faculty of Pharmacy, University of Ibadan, Ibadan, Nigeria; 3 Department of Biochemistry, Phytomedicine, Molecular Toxicology, and Computational Biochemistry Research Laboratory (PMTCB-RL), Bowen University, Iwo, Nigeria; 4 Department of Biotechnology and Food Science, Faculty of Applied Sciences, Durban University of Technology, Durban, South Africa; Teerthanker Mahaveer University, INDIA

## Abstract

**Background and objective:**

Traditionally, *Pteridium aquilinum* L. has been utilized as medicine for ages, however, it is not listed in the Nigerian herbal pharmacopeia, and there is no information regarding its standardization and antimicrobial activity. Therefore, the purpose of this study was to examine the pharmacognostic parameters and antimicrobial activity of *Pteridium aquilinum* leaf.

**Methods:**

Macroscopy, chemo-microscopy, fluorescence, and microscopic analyses of the leaf were investigated using standard methods. Qualitative and quantitative phytochemical screening, thin layer chromatography (TLC), GC-MS, and FTIR were also determined using standard procedures. Antioxidants were evaluated using DPPH. The antimicrobial activities of methanol extract and fractions were evaluated using Agar well diffusion method against *Candida albicans, Aspergillus niger, Staphylococcus aureus, Salmonella Typhimurium, Escherichia coli, and Pseudomonas aeruginosa*. The macroscopic features of *P. aquilinum* leaf include a bi-pinnate leaflet and alternate pinna arrangement. The GC-MS-identified compounds in the most active (DCM fraction) were docked against Candida albicans Sterol 14-alpha demethylase (5TZ1) and Escherichia coli DNA gyrase subunit B (6YD9).

**Results:**

The macroscopic features and microscopic features such as anomocytic stomata, numerous stomata in the abaxial layer, and absence of stomata in the adaxial layer were observed. Chemomicroscopy of the powdered leaves shows that the leaf contains tannins, starch, and lignin. GC-MS detected eighteen compounds. The antimicrobial test revealed that the dichloromethane fraction of *P. aquilinum* leaf was most active on all the test strains (bacteria and fungi) at 25 mg/mL to 100 mg/mL concentrations. Through *in silico* research, the binding of 1,2-benzenedicarboxylic acid, (4-hydroxybenzoyl) hydrazine, octadecadienoyl chloride, and 11,14-Eicosadienoic acid, detected in the DCM fraction by GC-MS analysis, to the active sites of 5TZ1 and 6YD9 was stable.

**Conclusion:**

This research gave scientific credence to the traditional medical practice of treating infections with *P. aquilinum* leaves.

## Introduction

Globally, microbial infections cause millions of deaths annually. About 10 million infections-related deaths (or 17%) were reported recently [1,2]. The growing prevalence of multidrug-resistant (MDR) bacteria concerns medical professionals and the pharmaceutical industry. This has led to a need for novel drugs that are affordable, accessible, and highly effective. Therefore, drug delivery systems, combination therapy with natural antibacterial substances, and the development of new antibiotic generations are all crucial [3,4]. There is a growing demand for the use of botanicals that have antimicrobial activity without endangering human health, and several botanicals have been worked on to validate their biological activities including antiviral, antioxidant, antibacterial, and anti-inflammatory properties [5–7].

Traditionally, medicinal plants have been utilized as medicines for ages [8]. Many still receive medical care from Traditional medicine practitioners, particularly in Africa. According to Chaachouay *et al*. [9], medications originating from medicinal plants or plant-derived formulations account for 25% of all medical prescriptions. According to the World Health Organisation (WHO), 80% of people globally still utilize medicinal plants to treat diseases. This is either because Western pharmaceuticals and healthcare are costly, or because traditional medicines are more pleasant to people from different cultural and religious backgrounds [10]. These plants’ therapeutic potential is derived from some secondary metabolites that have specific physiological effects on humans, including cardiac glycosides, anthraquinones, flavonoids, and polyphenols [11]. The increasing global trend towards using plants as a source of drugs has brought even more significance to natural drug discovery [12]. However, concerns regarding public health and the safety of therapeutic plants and products are growing along with their global use. There is relatively little information on the harmful effects of some herbal medicines, even though they are extensively used and have promising potential [13]. This is because the quality of these medicines is still untested and unknown. Inadequate labeling, adulteration, misidentification, inadequate quality controls, and subpar manufacturing processes jeopardize the safety of herbal products [14]. Therefore, it is important to standardize the quality and safety of these products. Furthermore, it is essential to provide appropriate information to the public as this will help them to better understand the hazards involved in using unstandardized herbal products [15].

*Pteridium aquilinum* L. existed before seed-producing plants evolved [16]. Through spores, they reproduce without flowering [17]. Woods, stream banks, and wet, shaded bottom areas are ideal habitats for *P. aquilinum* [18]. The fern flourish in locations with alluvial soil from rivers and streams that are moist but not wet. It may grow successfully in light to medium shade on upland soils with high levels of organic matter, and it can form dense colonies resistant to destruction by floodwaters [19]. This plant is highly effective in treating a variety of ailments, but not all parts of the plant have been investigated [20–23].

Furthermore, *P. aquilinum* is not listed in the Nigerian herbal pharmacopeia, and there is no information regarding its standardization. Owing to the resistance of microorganisms and the high cost of producing antimicrobial medications, it is necessary to identify and isolate novel antimicrobial compounds from medicinal plants. Therefore, the study aimed to carry out pharmacognostic evaluation and investigate the antimicrobial activity of *Pteridium aquilinum* leaves via *in vitro* and *in silico* studies.

## Materials and methods

### Materials and reagents

The materials used include powdered water bath (Gllenkamp, England), funnel (Pyrex, England), stirring rod ((Pyrex, England), weighing balance (Setra, England), 500 mL conical flask (Pyrex, England), Petri dishes (Pyrex, England), Distillation apparatus (quickfit, England), Rotary evaporator (Heidolph, Laborota 4000), pipette (Pyrex, England), the crucible (Pyrex, England), Measuring cylinder (Pyrex, England), beakers (Pyrex, England), test tubes (Pyrex, England), muffle furnace (Gllenkamp, England), microscope slides, cover slips, UV-vis spectrophotometer, syringes (Agar-jet, Agary, China), ruler, Cork borer, dissecting set, Autoclave (Gallenkamp, England), test tube racks, microscope (XSP- 103A), TLC Plate (Silica gel G60 F_254_ sheets 20 ×  20 cm, 0.5 mm thickness, Merck).

### Plant collection and authentication

The leaves of *Pteridium aquilinum* were collected from Abeokuta, Ogun State Nigeria. The plant was identified and authenticated by a taxonomist, Dr. Akinnibosun H.A, in the Department of Botany and Biotechnology, University of Benin, Benin City (UBH-B635) at University of Benin Herbarium (UBH), Benin City, Nigeria.

### Pharmacognostic evaluation

#### Macroscopical analysis.

Examined and described were the morphological traits of *Pteridium aquilinum*. The characteristics of the leaves, including their type, height, width, surface, taste, and odor, as well as the arrangement of leaves on the stem, petiole presence, lamina shape, apex, margin, base, venation, and texture, were noted and documented [24].

#### Qualitative microscopy.

Hand slices of the fresh leaf lamina were cut, put in a test tube with chloral hydrate, and boiled in a water bath for 4 h. The cleaned leaf sections were cooled, and then they were studied under a microscope to examine the surface features such as stomata, venation details, and type of epidermal cell [25].

#### Epidermal layers.

The translucent layers of abaxial and abaxial surfaces of the leaves were prepared using a razor blade. After that, the epidermal surfaces were cleaned by being submerged in a petri dish containing 5% sodium hypochlorite solution. After repeatedly rinsing the epidermal layers with water to get rid of the sodium hypochlorite, the tissue remnants were separated from the surfaces using a gentle Carmel hairbrush. Safranin O was applied to the epidermal surfaces for approximately two minutes, after which they were immersed in three different ethanol concentrations (50, 70, and 100%). A microscope slide of epidermal layers was covered with microscope slips after being mounted in glycerol. A light microscope was used to examine the slide. Using a clear camera, a photomicrograph of the features observed was captured at various magnifications [25].

#### Transverse section of leaf.

The midrib of the leaves was used to obtain the transverse section (T.S.), which was then stained with safranin and bleached with 5% sodium hypochlorite. The lamina was also prepared as a thin section. The photomicrographs of the stained sections were examined under a microscope were documented. Additionally, cellular diagnostic and diagnostic cell inclusions were identified in the powdered material [26].

### Proximate analysis

#### Determination of crude fiber.

200 milliliters of 1.25% sulfuric acid were heated with one gram of the defatted samples for thirty minutes in a beaker. Following a 30-minute boil with 200 mL of 1.25% sodium hydroxide, the boiled content is filtered, cleaned in hot distilled water, and then put back into the beaker. To neutralize the filtrates, they were rinsed with hot, distilled water. After being dried for 10–12 hours at 10–50 °C in a hot air oven, the crucible was cooled to a constant weight in a desiccator. After the crucible has burned all the organic material completely, it is placed in a muffle furnace and heated to 550–600 °C for two to three hours. It is then cooled in desiccators and tested for weight consistency [27]. The following formula was used to get the proportion of crude fiber:


$$\%{\text{Crude fibre = (W}}_{\text{1}}-{\text{W}}_{2}\text{)}\times \text{100}$$

Where W_1_ =  the crucible having crude fiber when cooled and weighed, and W_2_ =  the content of the crucible when ignited over a low flame until charred and then kept in a muffle furnace and weighed.

#### Determination of crude protein.

A 50 mL digestion flask containing approximately 5 g of dried plant materials was filled with 1 g of a digestion combination consisting of copper sulfate and sodium sulfate, and 15 mL of concentrated sulfuric acid was added for further breakdown. The mixture was heated until the frothing subsided, and the mixture turned clear. After gently boiling the mixture for an additional two hours, it was cooled and continuously stirred while being digested in 30 milliliters of water. The completed digest was poured into a 250 mL standard flask together with the necessary quantity of distilled water. Distillation equipment was used to carry out the process. Using one drop of methyl red as an indicator, 20 milliliters of 4% boric acid were measured. After that, 10 mL of the digested material was sent to the distillation assembly and combined with 20 mL of a solution containing 40% sodium hydroxide. The boric acid’s color changed from pink to blue in six minutes, signaling the end of the distillation process [27]. The boric acid having trapped the ammonia from the nitrogen of protein was titrated with 0.1N HCl, colour change from black to pink.

#### Determination of moisture content.

Weight measurements were taken of a warmed, tarred porcelain crucible with a cover (W1). After adding a spatula’s worth of the dried material to the crucible, the weight was measured again (W2). The sample was heated to 100 °C in an oven for 12 hours, with 6-, 3-, and 1-hour intervals until the weight remained constant. After cooling in a desiccator, the sample was weighed again. W3 and the constant weight were observed [28]. The following relationship was used to compute the % moisture:


$$\text{\%Moisture}content=\left[\frac{{\text{W}}_{2}-{\text{W}}_{3}}{{\text{W}}_{2}-{\text{W}}_{1}}\right]\times 100$$

Where the weight of the sample in the crucible is W_2_), the Constant weight is W3, the Weight of the sample in the crucible is W_2_, the Weight of the crucible is W1, W_2_ –W_1_ =  weight of the sample, and W_2_-W_3_ =  weight of the moisture-containing sample.

### Phytochemical analysis

The powdered leaf sample was subjected to phytochemical screening following conventional protocols to detect the presence of anthraquinones, flavonoids, terpenoids, alkaloids, and other compounds [27–29].

#### Extraction of plant material and solvent partitioning.

Two and a half (2.5 liters) of distilled methanol were used to extract nine hundred grams (900 g) of powdered *Pteridium aquilinum* leaf for 72 h while stirring continuously with a glass rod. This was repeated four times with 2.5 L of distilled methanol each. The liquid was then filtered using Whatman filter paper and a funnel. The marc was again macerated for 24 h with two liters of distilled methanol, and the filtrates were pooled and concentrated using a low-pressure rotary evaporator. After being chilled in desiccators, the extracts were kept in a freezer at 4 °C. Twenty grams of the methanol extract were partially re-dissolved in distilled water. After that, n-*n-hexane* was used to partition the solution and obtain the n-*n-hexane* portion. Dichloromethane and ethyl acetate were used to further partition the aqueous solution, leaving the aqueous fraction as the remaining aqueous phase. After being concentrated using a rotary evaporator and chilled in desiccators, the fractions were stored for later use.

#### DPPH free radical scavenging antioxidant activity.

From 200 µg/mL to 1000 µg/mL, the various fractions were produced. After adding around 1 mL of freshly made 1,1-diphenyl-2-picrylhydrazyl in methanol to 2 mL of each fraction, shaking the mixture briskly, and letting it sit in the dark for 30 minutes, the absorbance at 517 nm was measured. Ascorbic was used as the reference to calculate the absorbance of the control [30]. The free radical scavenging activity (FRSA) was calculated using the formula:


$$\text{\% FRSA}=\left[1-\left(\frac{\text{absorbance of control}}{\text{absorbance of sample}}\right)\right]\times 100$$

#### Determination of total phenolic contents.

The following calibration solutions for gallic acid were made in triplicate: 80 µg/mL, 120 µg/mL, 160 µg/mL, and 200 µg/mL. To prepare the reaction mixtures, 0.5 ml of various doses of gallic acid were added to 2.5 mL of sodium carbonate and 2.5 mL of Folin-Ciocalteu. With the use of a UV/visible spectrophotometer, the mixture was incubated for 30 minutes at room temperature to determine its phenolic content at 725 nm. Plotting absorbance versus concentration allowed for the construction of the calibration curve. The procedures were likewise carried out with samples at concentrations ranging from 200 to 1000 µg/mL, and absorbance was measured at 760 nm, accordingly. According to Ayeni *et al*. [31], the total phenolic content was measured in milligrams of gallic acid equivalent (GAE) per gram of sample.

#### Determination of total flavonoid contents.

A colorimetric test was used to assess the total flavonoid content [32]. 1.5 mL of methanol, 0.1 mL of 1 M potassium acetate, 0.1 mL of 10% aluminium chloride solution, and 2.8 mL of distilled water were combined with the sample, which ranged in concentration from 200 µg/ml to 1000 µg/ml in methanol. For thirty minutes, the combinations were let to stand at room temperature. The absorbance was measured with a UV/visible spectrophotometer at 415 nm. Concentrations of quercetin ranging from 200 µg/mL to 1000 µg/mL in methanol were used to produce the calibration curve. Quercetin equivalent (QE) milligrams per gram of fraction was the unit of measurement for the total flavonoid content, and the experiment was run in triplicate [33].

#### Thin layer chromatographic (TLC) fingerprint.

All the samples underwent a one-way ascending approach thin-layer chromatography profile using TLC pre-coated plates (silica gel 60). At 105 °C, the plate was activated in the oven. The plates were scored at 0.5 cm from the side and 1 cm from the bottom using a pencil and scissors. Every fraction of *Pteridium aquilinum* was made, slightly dissolved in the suitable solvent, and then the dissolved samples were evenly applied to the plates using capillary tubes, separated by 0.5 cm, and left to dry. Using a new solvent system—*n-hexane*: ethyl acetate at a 4:1 ratio—the plates were produced in a chromatographic tank. After being air-dried and exposed to daylight and UV light at 254 and 365 nm, the produced TLC plate was observed. A picture of the chromatograph was taken. Using the following formula, the R_f_ value for every spot was determined. R_f_ is equal to a/b, where a is the distance from the application point to the spot’s center and b is the distance from the solvent front to the application point.

#### Antimicrobial assay.

The microorganisms used for this assay were all collected from the Department of Medical Microbiology Laboratory, University of Ibadan, Ibadan, Nigeria. The bacteria isolates were *Salmonella typhi* (ATCC 14028), *Staphylococcus aureus* (ATCC 29213), *Pseudomonas aeruginosa* (ATCC 27853), and *Escherichia coli* (ATCC, 25922) while the fungal isolates were *Aspergillus niger* and *Candida albicans* (both clinical isolates). The bacteria isolates were sub-cultured in nutrient agar while the fungi isolates were sub-cultured in Sabouraud dextrose agar.

#### Preparation of media for antimicrobial assay.

Mueller-Hinton Agar (40 g) and Sabouraud (68 g) were weighed and thoroughly mixed with a swirling motion in 1000 mL of distilled water. The mixture was then autoclaved for 15 minutes at 121 °C, let to cool to 47 °C, and then mixed again before being poured onto a sterilized petri dish in 10 mL. Additionally, 1000 milliliters of distilled water were weighed with thirty grams (30 g) of dehydrated medium. Stirring often, heat until it boils for one minute. Sterilise for fifteen minutes at 121 °C [34].

#### Antibacterial assay.

The agar diffusion method [35] was employed for the antibacterial screening. All extracts/fractions were dissolved in 10% dimethylsulfoxide (DMSO) to give 100 µg/mL stock from where a two-fold dilution was prepared to give five 25 µg/mL, 50 µg/mL, and 100 µg/mL. A 20 mL volume of sterile Mueller-Hinton agar was poured into sterile petri dishes and allowed to solidify after which 0.2 mL inoculum of 0.5 MacFarland solution of the bacterial strains was spread using sterile swabs on the solidified petri dishes using sterile swabs and left for 5 min on bench. Wells were bored in the Petri dishes using a 6 mm diameter sterile cork borer and were filled with the different concentrations of each extract/fraction while 10% DMSO and gentamycin were used as negative and positive controls, respectively. They were left on the bench for 30 min to allow for diffusion of the extracts/fractions after which they were incubated at 37^0^C for 24 h. The assay was done in triplicates, the diameter of the zones of inhibition was measured and the mean was calculated.

#### Antifungal assay.

This experiment was carried out using the Agar diffusion method [36]. The Crude extract and all fractions were dissolved in 10% dimethylsulfoxide to give 100µg/mL stock from which a 2-fold dilution of extracts/fractions was prepared to give 25 µg/mL, 50 µg/mL, and 100 µg/mL concentrations. A 0.2 mL standard inoculum of the fungal strains was dispersed in tryptone soya broth and spread on Petri dishes containing 20 mL sterile Sabouraud dextrose agar (SDA) using sterile swabs, and they were left on the bench for 5 min. A 6 mm diameter sterile cork borer was used to bore wells which were filled with the different concentrations of the extracts/fractions, while ketoconazole and 10% DMSO were used as positive and negative controls, respectively. The whole setup was done in triplicates. They were left on the bench for about 30 min to allow for diffusion and then incubated at 25 °C for 24 h after which the diameters of zones of inhibition were measured.

#### Determination of minimum inhibitory concentration (MIC) and minimum bactericidal concentration (MBC).

The lowest concentration of an antimicrobial agent that can stop the growth of a microorganism after it has been incubated for around 24 h is known as the minimum inhibitory concentration, and the lowest concentration known as the minimum bactericidal concentration will stop an organism’s growth after it has been subcultured into media free of antimicrobials [37].

#### Macrodilution broth method.

Eight labeled microtiter plates were prepared with Tryptic soy broth (TSB) or Trypticase soy broth (TSB) by double-fold serial dilution of plant fractions (n-*n-hexane*, dichloromethane, ethylacetate, crude and aqueous), along with Gentamycin (positive control), to achieve a concentration ranging from 50 µg/mL to 1.563 µg/mL. A 100 µ L standardized inoculum was added to each dish, and the infected plates were then incubated for 24 hours at 37 °C. Gentamycin and ketoconazole were used as the positive control, whereas plates with only seeded broth served as the negative control. The minimal inhibitory concentration (MIC) was defined as the lowest concentration at which, in comparison to the control, no discernible growth was possible.

#### Minimum inhibitory concentration (MIC) and minimum bactericidal concentration (MBC).

The minimum inhibitory concentration is the lowest concentration of antimicrobial agent that can inhibit the growth of microorganisms after incubation for about 24 hours, while the minimum bactericidal concentration is the lowest concentration of antimicrobial agent that prevents the growth of bacteria in antimicrobial- free media [38]. Micro-broth dilution method was used. A two-fold serial dilution of plant fractions (n-*n-hexane*, dichloromethane, ethylacetate) and extract were prepared in tryptone soya broth (TSB) in 96-well microtiter plates, to give graded concentrations from 50 µg/mL to 1.563 µg/mL. Each well was seeded with 100 µ L of the standardized inoculum of the bacterial and fungal isolates and the inoculated plates were incubated at 37 °C for 24 h. The set of wells seeded with only broth served as negative control while the set of wells containing gentamycin and ketoconazole served as positive controls for the bacterial and fungal isolates, respectively. The lowest concentration that did not permit any visible growth when compared with control was considered as the minimum inhibitory concentration (MIC). The MBC was determined by a modification of the method of [39]. A 100 µ L aliquot from the MIC and sub-MIC tubes was placed and spread uniformly on extract-free Muller-Hinton agar plates. The plates were incubated at 37 ºC for 24 h and thereafter examined for growth. The lowest concentration for each extract that prevented bacterial growth on extract-free agar after 24 h at 37 °C of incubation was recorded as the minimum bactericidal concentration (MBC).

#### Fourier transform infrared spectroscopy (FTIR) spectral studies.

A Fourier Transform Infrared Spectroscopy (FTIR) spectrometer (Model FTIR-8400S SHIMADZU) was used to determine the structures of the chemicals found in the dichloromethane fraction of *Pteridium aquilinum*. For the FTIR study, the plant sample’s ethanol extract was utilized. The sample was dried overnight at 120 °C in a hot oven. A translucent sample disc was prepared by encapsulating approximately 10 mg of the sample in 100 mg of ATR pellet. An FTIR spectroscope (Model FTIR-8400S SHIMADZU 64 scans) was used to load the plant extract of ethanol. At a resolution of 4 cm^-1^, the spectrums were obtained using wavelengths ranging from 4000 cm^-1^ to 400 cm^-1^ used to quantify infrared values [40].

#### GC-MS identification of chemical constituent of dichloromethane fraction.

The GCMS (QP2010 PLUS SHIMADZU) was used to perform the GC-MS analysis of dichloromethane fraction (most active fraction) of *Pteridium aquilinum*. A 95% dimethyl polysiloxane capillary column with dimensions of 30 mm by 0.25 mm and a film thickness of 0.25 mm was utilized as the column. It was manufactured by Perkin Elmer. Helium was utilized as the carrier gas, with a flow rate of 0.5 mL/min. The sample injection volume used was one microliter (1 μL). A constant 250 °C was maintained at the entrance. Initial programming for the oven temperature was set at 80 °C for 4 minutes, followed by increases to 200 °C and 280 °C at a rate of 20 °C per minute, with a 5-minute rest period. There was a 45-minute runtime. The temperature of the MS transfer line was kept constant at 200 °C. At 180 °C, the source temperature was kept constant. To identify and quantify the compounds, total ion count (TIC) was used to analyze the data from the GC-MS analysis, which was conducted using electron impact ionization at 70 eV. Each constituent’s spectrum was compared to the database of known component spectrums kept in the GC-MS library [27]. To identify the separated components, they were converted into mass spectra peaks and compared to mass spectra chromatograms in the integrated GC–MS library software from the National Institute of Standards and Technology (NIST). A library spectrum search is conducted by NIST using the mass spectrometer detectors’ (MSD) reported unknown spectrum. A hit list of compounds with chemical structures similar to the sample compounds is generated based on the degree of confidence. A compound was chosen from the hit list based on a GC-MS analysis probability score of at least 50% matching from the NIST library. An indicator of how closely a compound in the sample provided in a peak in a GC-MS analysis resembles recognized compounds in the NIST library is the NIST library probability score [41].

### *In silico* studies

#### Retrieval and preparation of proteins.

Candida albicans Sterol 14-alpha demethylase in conjunction with oteseconazole (PDB ID: 5TZ1) is taken from the Protein Data Bank (PDB) [42] as well as the compound N-[6-(3-azanylpropanoylamino)-1,3-benzothiazol-2-yl]-3,4-bis(chloranyl)-5-methyl-1H-pyrrole-2-carboxamide (ON2) formed by Escherichia coli DNA gyrase subunit B.PDB ID: 6YD9 [43]. The protein’s co-crystallized ligand molecules and crystallographic water were removed, and MGL-AutoDockTools (ADT, v1.5.6) were used to add the lost hydrogen atoms. The partial atomic charge was substituted with the Kollamn charges [44].

#### Ligands preparation.

The dichloromethane fraction’s Structure Data Format (SDF) for the GCMS and the co-crystallized compounds were evaluated using the PubChem database (www.pubchem.ncbi.nlm.nih.gov). Openbabel was used to reduce the chemicals [45]. According to Trott and Olson [46], the ligands were converted to PDBQT format using the AutoDock function capabilities included in PyRx 0.8.

### Molecular docking studies

#### Active site-directed molecular docking of identified compounds to target proteins.

Before the compounds were docked to the enzymes, the protocol for the molecular docking analysis procedure was verified through validation. After being removed from the crystallized structure, the native ligands were re-docked to the protein’s binding site. The native ligand was superimposed with the chosen conformer of the ligands from the docking analysis that had the best pose, and the RMSD was then calculated using Discovery Studio. Docking of the reference inhibitors and the compounds discovered by GCMS to the active sites of 5TZ1 and 6YD9 was done using AutoDock Vina in PyRx 0.8 [46]. After being imported into PyRx 0.8 using OpenBabel, the ligands were reduced to a minimum [45]. Conjugate slope descent was used in the optimization procedures, and the Universal Force Field (UFF) was used in the energy minimization parameter. The dimensions of the enzymes’ binding sites were determined by analyzing the size and center of the grid boxes. The default mode was used in every docking procedure. The centroid of the co-crystallized ligand bind site on the produced protein, the coordinates of which were given in S1 Table in S1 File, was surrounded by a cubic grid box.

## Results

### Pharmacognostic evaluation of *Pteridium aquilinum* leaf

#### Macroscopic evaluation of *P. aquilinum* leaf.

S2 Table in S1 File displays the macroscopic properties of the powdered leaf and leaf of *P. aquilinum*. The leaf is light green in color, odorless, and tasteless. Its macroscopic and organoleptic characteristics showed that each pinna has deep cuts that stop short of the central vein, the leaf is smooth, and the leaf apex is truncated. The leaflet is bipinnate and arranged alternately with the rachis. The venation pattern is furcated, and the leaf border is whole. The leaf powder is a brownish hue and has no flavor or smell.

#### Histology of *Pteridium aquilinum* leaf.

**Fig 1:**
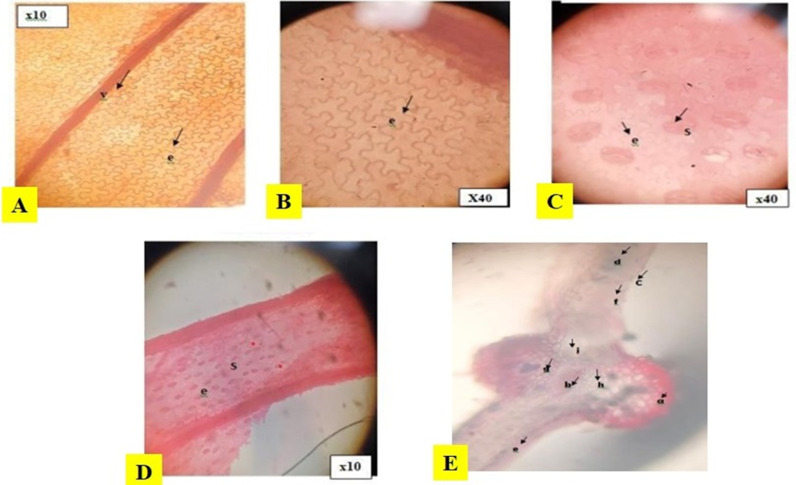
The Adaxial epidermal feature (A and B), Abaxial epidermal features (C and D), and Transverse section (E) of P. aquilinum leaf. a: Collenchyma, b: pith, c: Upper epidermis, d: Border sclerenchyma, e, and f: Lower cylindrical palisade cells, g: Phloem, h: Xylem, i: Respiratory cavity, v: Venation pattern e: Epidermal cell, s: Stomata.

Fig 1 displays the results of the histological analysis of the *P. aquilinum* leaf. It reveals that the abaxial layer of the epidermal layer has many anomocytic stomata, wavy epidermal cells, and a venation pattern, while the adaxial layer lacks stomata and has wavy epidermal cells (S3 Table in S1 File). Fig 1e displayed the transverse section of *P. aquilinum*. The features of this structure include a collenchyma, with upper epidermis, border sclerenchyma, lower cylindrical palisade cells, phloem, xylem, and respiratory cavity.

#### Microscopic evaluation of *P. aquilinum* leaf.

S4 Table in S1 File displays the microscopically evaluated powdered leaf. The outcome showed that trichomes were absent but epidermal cells, fiber, and vasculature were there. The identification and standardization of *P. aquilinum* can be aided by these criteria.

#### Fluorescence analysis of *P. aquilinum.*

S5 Table in S1 File contains the results of the fluorescence analysis observed under daylight and UV light. The fluorescence results for the *P. aquilinum* leaf powder are presented as follows: picric acid: daylight (yellow), UV-254 nm (light green), 365 nm (purple); Fecl_3_:daylight (brown), UV-254 nm (light green), 365 nm (purple), acetic acid: daylight (brown), UV-254 nm (light green), 365 nm (yellow), 50% HCl: daylight (brown), UV-254 nm (light green), ^365 nm (purple); 50% H^_2_^SO^_4_:^daylight (light green), UV-254 nm (green), 365 nm (purple);^ methanol: daylight (light green), UV-254 nm (green), 365 nm (red); ethanol: daylight (light green), UV-254 nm (green), 365 nm (light green).

#### Chemomicroscopic character of dried leaf powder of *P. aquilinum.*

S6 Table in S1 File displays the dry leaf powder chemo-microscopy characteristics of *P. aquilinum*. When N50 iodine was added to the powder, a blue-black color was seen, indicating the presence of starch. When Sudan red IV was applied, a pink-red hue was seen. The addition of Ruthenium red to the dried powder proved the presence of mucilage, which had a red coloration. When HCl was added, no crystals were visible, indicating that there was no calcium oxalate present.

#### Proximate parameters analysis of *P. aquilinum* leaf powder.

S7 Table in S1 File provides a summary of the approximate analysis of the dried powder leaf of *P. aquilinum* in percentage. The results show that the moisture content was 11.15 ± 0.50%, the crude protein was 0.83 ± 0.03%, the crude fat was 0.44 ± 0.24%, the crude fiber was 45.64 ± 0.21%, and the crude carbohydrates was 33.04 ± 0.02%.

#### The percentage yield of extract of *P. aquilinum* leaf.

S8 Table in S1 File displayed the percentage yield of 2.06% for the methanol extract of *P. aquilinum* leaf.

#### Phytochemical screening of *P. aquilinum* leaf extract.

**Fig 2:**
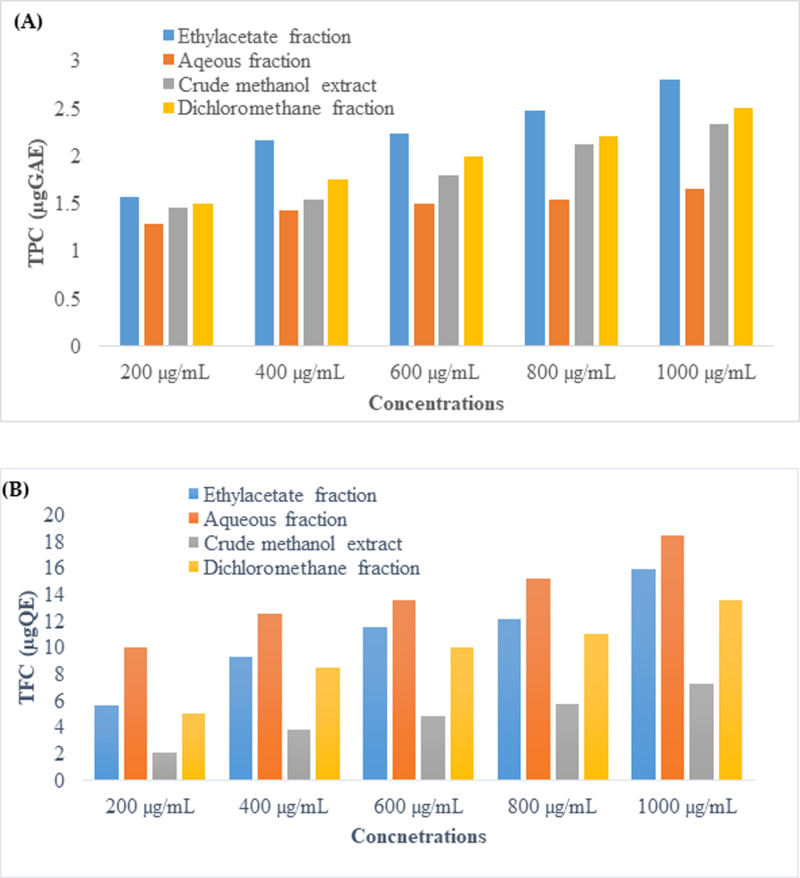
Total phenol contents (A) and total flavonoid contents (B) of *Pteridium aquilinum* solvent fractions and crude extract.

S9 Table in S1 File displays the results of the qualitative phytochemical study conducted on the *P. aquilinum* leaf fractions. The findings indicated that anthraquinones, alkaloids, and terpenoids are present in the n-*N-hexane* fraction; flavonoids, anthraquinones, alkaloids, and terpenoids are present in the DCM fraction; flavonoids, cardiac glycosides, alkaloids, and terpenoids are present in the ethyl acetate fraction; and saponin, tannins, flavonoids, anthraquinones, alkaloids, steroids, and terpenoids are present in the methanol fraction and crude extract. Fig 2 displays the findings for the quantitative phytochemicals found in the leaves of *P. aquilinum*. The findings showed that, as shown in Fig 2A, the ethyl acetate fraction had the highest total phenol content, followed by the crude extract and methanol fraction. In contrast, Fig 2B showed that the methanol fraction had the highest total flavonoid content, followed by the ethyl acetate fraction and the crude extract.

#### Antioxidant evaluation.

**Fig 3:**
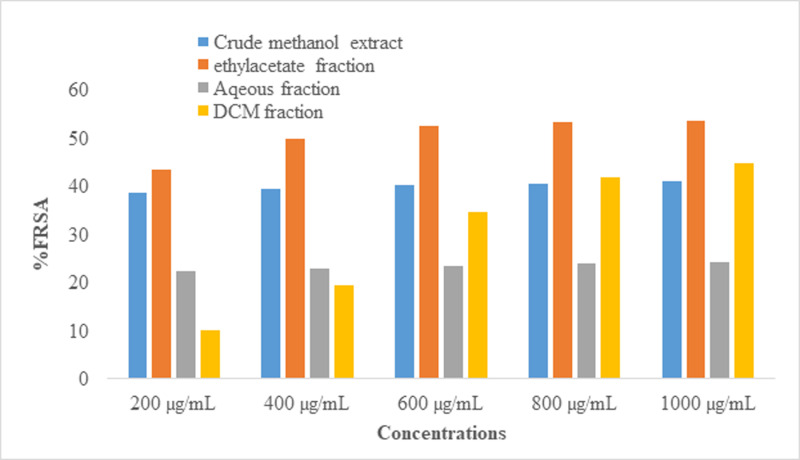
DPPH scavenging activity of crude and fractions of *P. aquilinum.*

Fig 3 illustrates how the antioxidant activity of *P. aquilinum* leaf crude extract and fractions were evaluated using DPPH at concentrations of 200 µg/mL, 400 µg/mL, 600 µg/mL, 800 µg/mL, and 1000 µg/mL, respectively. According to the findings, the fractions containing ethyl acetate, dichloromethane, and methanol have the strongest antioxidant activity.

### Phytochemistry

#### Thin layer chromatography.

Pre-coated plates were used for thin-layer chromatography (TLC). Before the development of the ethanol extract on the plate, it was activated for thirty minutes. Utilizing the UV lamp (254 nm and 365 nm) allowed for the detection of the secondary metabolites. Table 1 and S1 Fig in S1 File show the results of the calculation of the R_f_ value based on the number of spots observed.

**Table 1:** Thin layer chromatography of screening of *P. aquilinum* leaf.

Samples	Daylight			UV Lamp					
				**254 nm**			**365 nm**		
	**S**	**Color**	**R**_**f**_	**S**	**Color**	**R**_**f**_	**S**	**Color**	**R**_**f**_
HF	5	Green	0.16	6	Green	0.05	8	Blue	0.05
		Green	0.26		Green	0.16		Pink	0.16
		Green	0.44		Green	0.29		Pink	0.29
		Green	0.55		Green	0.44		Pink	0.44
		Yellow	0.96		Green	0.55		Pink	0.55
		–	–		–	–		Blue	0.72
		–	–		–	–		Light blue	0.90
		–	–		–	–		Deep pink	0.97
DF	4	Green	0.18	5	Green	0.18	6	Pink	0.18
		Green	0.30		Green	0.30		Pink	0.30
		Green	0.45		Green	0.47		Pink	0.47
		Green	0.57		Green	0.57		Pink	0.57
					Green	0.91		Blue	0.75
								Pink	0.86
EF	1	Green	0.45	1	Purple	0.91	1	Green	0.45
CF	3		0.21	4	Green	0.21	4	Pink	0.21
			0.39		Green	0.39		Pink	0.39
			0.49		Green	0.52		Pink	0.52
					Green	0.71		Blue	0.71

**S:** No of spots, **R**_**f**_: Retention factor, **E:** Ethyl acetate **H:** *N-hexane*, **HF:** *N-hexane* fraction, **DF**: dichloromethane, **EF**: Ethyl acetate, **CF**: Crude extract. Solvent system =  E1:H4

#### Antimicrobial activity of crude extract and fractions of *P. aquilinum* leaf.

**Fig 4:**
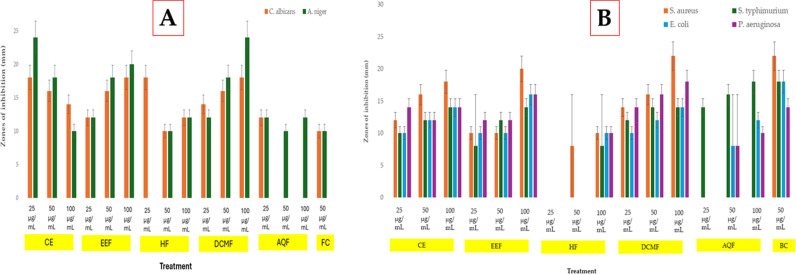
Anti-fungal activity (A) and Anti-bacterial activity (B) of crude extract and fractions of *P. aquilinum.* FC: Fungi control (ketoconazole); BC: Bacterial control (gentamycin).

Results of the investigation into the antibacterial properties of *P. aquilinum* leaf fractions and crude extract are displayed in Fig 4 (S11 and S12 Tables in S1 File) and Table 2. All extracts and fractions showed activity against *C. albicanss* and *A. niger* (except the aqueous fraction that showed no activity against *C. albicanss*) with the DCM fraction having the most notable activity at 25-100 mg/mL concentrations having zones of inhibition that are comparable to that of ketoconazole. Dichloromethane fraction (DCMF) at all concentrations inhibited the growth of the bacterial isolates with zones comparable to that of Gentamycin. At 25 mg/mL and 50 mg/mL, DCMF exhibited higher activity against the bacterial isolates than the crude extract (CE)*,* ethyl acetate (EAF), *n-hexane* (HF), and aqueous (AQF) fractions. DCMF gave an MIC range of 1.56 to 12.50 mg/mL and an MBC range of 1.56 to 25 mg/mL, both of which were comparable to the values obtained for the drug control (Gentamycin). Also, for the fungal isolates, DCMF gave MIC and MBC values ranging between 6.25 and 12.5 mg/mL which favorably compares with values obtained for the drug control, Ketoconazole.

**Table 2:** Minimum inhibitory concentration and minimum bactericidal/fungicidal inhibitory concentrations of crude extract and fractions of *P. aquilinum.*

Isolates	Zones of inhibition produced by different antibiotics (mm)											
	**AQF**		**EAF**		**DCM**		**CE**		**BC**		**FC**	
	**MIC**	**MBC**	**MIC**	**MBC**	**MIC**	**MBC**	**MIC**	**MBC**	**MIC**	**MBC**	**MIC**	**MBC**
*S. aureus*	1.25	12.5	25	25	1.56	1.56	3.13	>50	1.25	5	NA	NA
*S. tyhi*	25	25	25	25	12.5	12.5	12.5	25	>20	>20	NA	NA
*E. coli*	12.5	12.5	25	25	6.25	25	3.13	6.25	2.5	>10	NA	NA
*P. aeruginosa*	25	25	25	25	12.5	12.5	>50	>50	5	>10	NA	NA
*C. albicans*	25	>50	50	50	12.5	12.5	50	>50	NA	NA	5	5
*A. niger*	12.5	>50	25	25	6.25	6.25	25	>50	NA	NA	10	>10

Key: CE: Crude extract, EAF: Ethyl acetate fraction, HF: *N-hexane* fraction, DCMF: Dichloromethane fraction, AQF: Aqueous fraction BC: Bacterial control Standard drug (gentamycin), FC: Fungi control (ketoconazole

### *In silico* analysis

#### Validation of molecular docking protocol.

**Fig 5:**
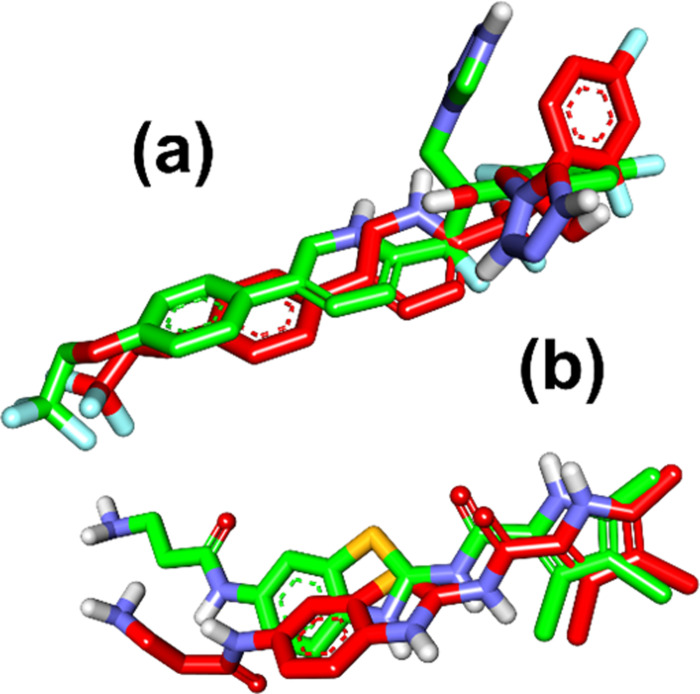
Superimposed docked conformer of the native ligand on the extracted conformation of (a) sinefungin and (b) safinamide. Green: co-crystalized ligand conformer and red: selected docked conformer.

Authenticating the molecular docking approach is a crucial stage in the virtual screening of compounds used in the protocol to predict the accuracy and precision of the docking process [47]. An early validation was conducted on the docking methodology that will be used for the docking steps. Following their extraction, the target protein’s co-crystallized ligands (5TZ1 and 6YD9) were superimposed and aligned with the least energy conformer on the chosen docked poses (Fig 5). For the compounds oteseconazole and ON2301, the RMSD was determined using BIOVIA Discovery Studio Visualizer 20.1.0, San Diego, CA, USA 2020, and was 3.023 and 2.255 Å, respectively (Fig 2).

#### FTIR analysis dichloromethane fraction *P. aquilinum* leaf.

The dichloromethane fraction of *P. aquilinum* leaf (which is the most active fraction) was subjected to FTIR spectral analysis, which revealed diagnostic peaks of several functional groups on FTIR spectra. These peaks include 1026.16 (C-N stretching), 1103.32 (C-O stretching), 1195.91 (C-O stretching), 1257.63 (C-O stretching), 1404.22 (O-H bending), 1705.13 (C=O stretching), 2036.9 (N=C=S stretching), 2345.52 (O=C=O stretching), 2538.41 (O-H stretching), 2831.6 (N-H stretching), 2947.33 (C-H stretching), 3348.54 (O-H stretching), 3742.03 (O-H stretching), and 3873.19 (O-H stretching) (S10 Table S1 File; S2 Fig in S1 File).

#### GC-MS analysis of dichloromethane fraction of *P. aquilinum* leaf.

As shown in Table 3 (S3 Fig S1 File), GC-MS analysis was used to determine the active components of dichloromethane fraction *P. aquilinum* leaf. A total of eighteen [17] compounds with varying retention times (RTs) were discovered. The results showed the presence of benzoic acid, 3-hydroxy-, methyl ester, 4-decyl methylphosphonofluoridate, dipentene diepox, palmitic acid, methyl ester, palmitic acid, linolelaidic acid, methyl ester, 13-octadecenoic acid, methyl ester, stearic acid, methyl ester, z-9-tetradecena, pentanoic acid, palmitin, 1,2-di-, 2-aminoethyl hydrogen phospha, adipic acid, bis(2-ethylhexyl) ester, linoleic acid chloride, cis-oleic acid, stearic acid diglycer, phthalic acid, dioctyl ester, cis-9-hexadecenal and linoleic acid chloride. Z-9-Tetradecena ester has the highest content of 24.99% and the 4-Decyl methylphosphonofluoridate contains the lowest content of 0.88%.

**Table 3:** GC-MS analysis result of *P. aquilinum* leaf dichloromethane fraction.

S/N	R.T.	Compound	Molecular weight	Base Peak Area (%)
1.	11.042	(4-Hydroxybenzoyl) hydrazine	152	10.27
2.	12.917	4-Decyl methylphosphonofluoridate	238	8.41
3.	14.575	7-Oxabicyclo[4.1.0]heptane	168	3.65
4.	15.725	Hexadecanoic acid	270	6.28
5.	17.083	n-Hexadecanoic acid	256	3.48
6.	18.950	11,14-Eicosadienoic acid	322	4.67
7.	19.008	13-Octadecenoic acid	296	4.67
8.	19.367	Eicosanoic acid	326	6.28
9.	20.183	cis-11-Hexadecenal	238	4.67
10.	21.075	(E)-13-Docosenoic acid	338	4.67
11.	21.717	1-[[[(2-aminoethoxy)hydroxyphosphinyl]oxy]methyl]-1,2-ethanediyl ester	691	4.84
12.	22.742	*N-hexane*dioic acid	370	4.84
13.	23.125	9,12-Octadecadienoyl chloride	298	4.67
14.	23.592	Oleic Acid	282	4.67
15.	23.800	Octadecanoic acid	624	4.84
16.	23.800	1-Fluorononane	146	4.84
17.	24.308	1,2-Benzenedicarboxylic acid	390	4.84
18.	25.383	7-Tetradecenal	210	4.67
19.	25.742	9,12-Octadecadienoyl chloride	298	4.67

#### Molecular docking study.

Eighteen compounds and reference compounds (S1 and S2) were discovered by the molecular docking of the GCMS against 5TZ1 and 6YD9. The binding energy ranges for 5TZ1 and 6YD9, respectively, are -7.3 to -4.3 Kcal/mol and -6.4 to -4.3 Kcal/mol. S1 and S2, the reference compounds, have binding energies of -10.8 and -8.1 Kcal/mol for the 5TZ1 and 6YD9 enzymes, respectively. The selection of the top two compounds was based on their interaction with the catalytic residues of the respective proteins, docked poses, and binding scores. The top two compounds to the 5TZ1 were 11,14-Eicosadienoic acid and 9,12-Octadecadienoyl chloride, with binding energies of -7.2 and -7.3 9.3 kcal/mol, respectively; the top two compounds to the 6YD9 were 1,2-Benzenedicarboxylic acid (4-Hydroxybenzoyl) hydrazine, with a binding energy of -6.4 kcal/mol (Table 4).

**Table 4:** Binding energies of the GCMS identified compounds from dichloromethane fraction docked in the active sites of Candida albicans Sterol 14-alpha demethylase (5TZ1) and Escherichia coli DNA gyrase subunit B (6YD9).

S/No	Ligand	Binding Affinity (Kcal/mol)	
**5TZ1**	**6YD9**
	Oteseconazole (E = 528.81)	−10.8	
S2	ON2 (E = 597.52)		−8.1
1	9,12-Octadecadienoyl chloride (E = 57.94)	−7.3	−5.7
2	11,14-Eicosadienoic acid (E = 75.18)	−7.2	−5.9
3	(E)-13-Docosenoic_acid (E = 120.29)	−7.1	−5.9
4	13-Octadecenoic_acid (E = 65.81)	−6.9	−5.9
5	Oleic_Acid (E = 81.73)	−6.7	−5.9
6	Eicosanoic_acid (E = 82.09)	−6.7	−5.6
7	Hexadecanoic_acid (E = 57.46)	−6.6	−5.9
8	cis-11-Hexadecenal (E = 108.83)	−6.6	−5.9
9	1,2-Benzenedicarboxylic acid (E = 151.34)	−6.5	−6.4
10	n-Hexadecanoic_acid (E = 57.46)	−6.5	−5.8
11	7-Tetradecenal (E = 47.55)	−6.5	−5.6
12	Octadecanoic_acid[35] (E = 66.36)	−6.5	−5.5
13	1-2-aminoethoxyhydroxyphosphinyl]oxy]methyl-1,2-ethanediyl_ester (E = 767.86)	−6.5	−4.9
14	(4-Hydroxybenzoyl)hydrazine (E = 103.33)	−6.1	−6.4
15	4-Decyl_methylphosphonofluoridate (E = 314.73)	−5.9	−5.4
16	1-Fluorononane (E = 24.87)	−5.2	−4.9
17	*N-hexane*dioic_acid (E = 33.38)	−5	−5.4
18	7-Oxabicyclo[4.1.0]heptane (E = 1512.64)	−4.3	−4.3

#### Amino acid interactions of top docked compounds from dichloromethane fraction.

**Fig 6:**
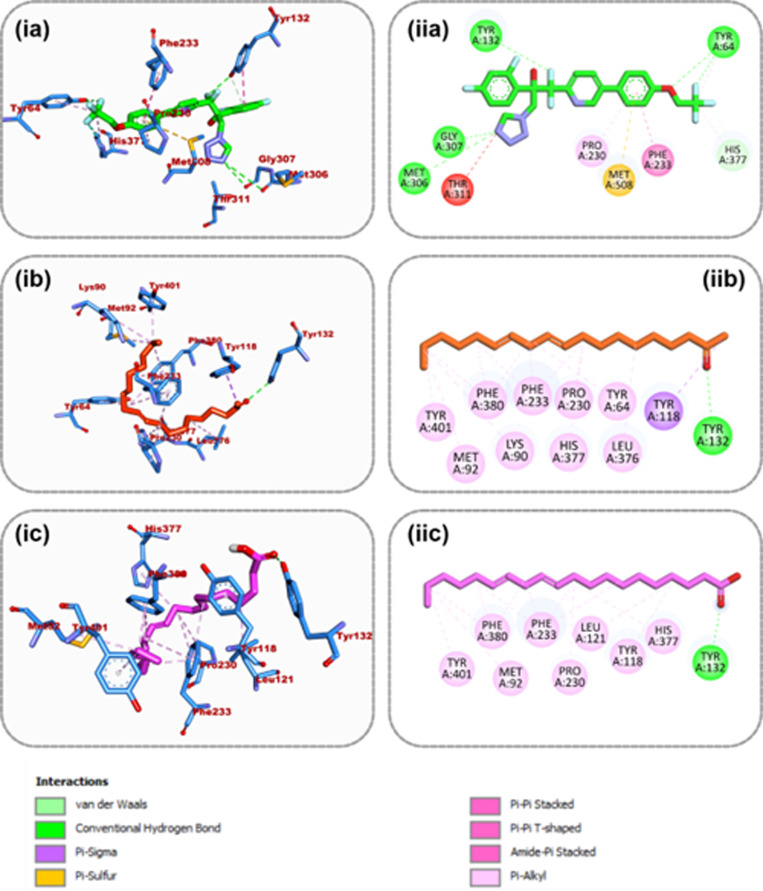
Amino acid interactions of top three GCMS identified compounds from dichloromethane fraction and reference inhibitor in the active site of 5TZ1 (i) 3D representation (ii) 2D representation. The ligands are presented in stick representation as shown in colors: (a) green: Oteseconazole (b) Orange: 9,12-Octadecadienoyl_chloride (c) purple: 11,14-Eicosadienoic_acid.

**Fig 7:**
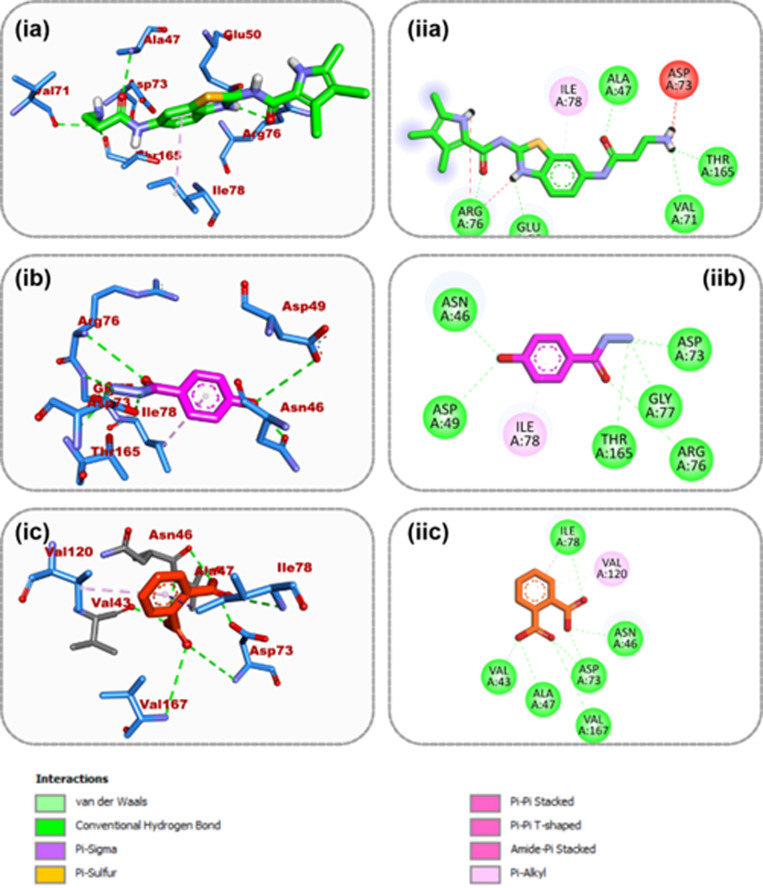
Amino acid interactions of top three GCMS identified compounds from dichloromethane fraction and reference inhibitor in the active site of 6YD9 (i) 3D representation (ii) 2D representation. The ligands are presented in stick representation as shown in colors: (a) green: ON2 (b) purple: 1,2-Benzenedicarboxylic_acid (c) (4-Hydroxybenzoyl) hydrazine.

Table 5 lists all the binding interactions that occur in the target proteins’ active sites. Based on the interactive analysis, it was possible to successfully re-dock Oteseconazole, the co-crystallized ligand of 5TZ1, in the protein’s active region. Several ligand groups of oteseconazole formed four typical hydrogen bonds with the amino acid residues Met306, Gly207, Tyr132, and Tyr64 of the enzyme’s active site. With Met508, Phe233, and Pro230, a pi-sulfur, pi-pi T-shaped, and pi-alky contact was established (Fig 6a). Tyr132, Tyr118, and many alkyl connections were generated by the top-docked chemical, 9,12-Octadecadienoyl chloride, to the same protein (Fig 6b). At the 6YD9 active site, ON2 connected with six hydrogen atoms at Asn45, Asp49, Thr165, Gly77, Arg76, and Asp73 in addition to one pi-alkyl contact at Ile78 (Fig 7c). A pi-alkyl contact with Val120 and six hydrogen bonds with Asn46, Asp73, Ala47, Val43, Val167, and Ile78 were formed by 1,2-Benzenedicarboxylic acid, the top docked molecules to 6YD9 (Fig 7b). The second top-docked molecule to 6YD9, (4-Hydroxybenzoyl) hydrazine, also formed a pi-alkyl contact with Ile78 and five hydrogen bonds with Asp49, Asn46, Thr165, Gly77, Arg76, and Asp73 (Fig 7c).

**Table 5:** Amino acid interactions of *human* catechol O-methyltransferase (hCOMT) and *human* monoamine oxidase B (MOA) with the reference inhibitors and top three docked compounds from the docking analysis.

	Protein	Hydrogen bonds			Hydrophobic Interaction
**Numbers**	**Interacted residues**	**Numbers**	**Interacted residues**
Oteseconazole	5TZ1	4	Tyr132 Gly307 Met306 Tyr64	3	Pro230 Met508 Phe233
9,12-Octadecadienoyl chloride		1	Tyr132	10	Tyr401 Phe380 Phe233 Pro230 Tyr64 Tyr118 Met92 Lys90 His377 Leu375
11,14-Eicosadienoic acid		1	Ser119 Gln120 Ile89	3	Tyr401 Phe380 Phe233 Pro230 Tyr64 Tyr118 Met92 His377 Leu121
ON2	6YD9	5	Arg76 Glu50 Ala47 Thr165 Val71	1	Ile78
1,2-Benzenedicarboxylic acid		7	Val43 Ala47 Val167 Asp73 Asn46 Ile78	I	Val120
(4-Hydroxybenzoyl) hydrazine		6	Asn46 Asp49 Asp73 Gly77 Thro165 Arg76	1	Ile78

Human catechol O-methyltransferase (hCOMT); human monoamine oxidase B (hMOA)

#### Molecular docking.

**Fig 8:**
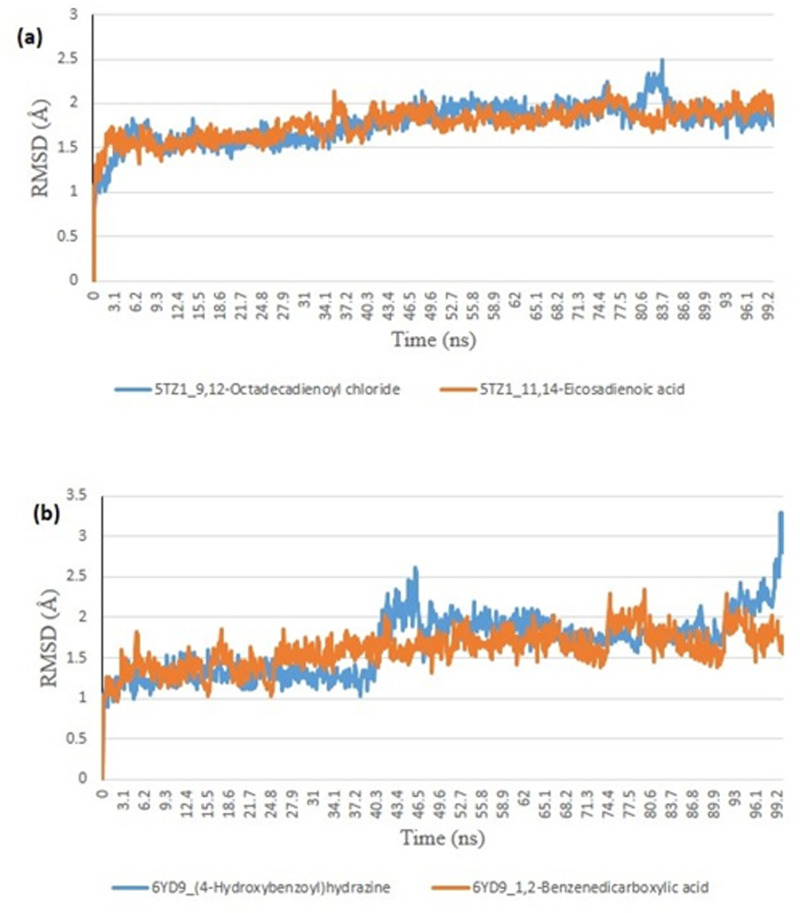
The backbone-root mean square deviation (RMSD) plots of molecular dynamics (MD) simulation of ligands complexed to (a) 5TZ1 (b) 6YD9.

**Fig 9:**
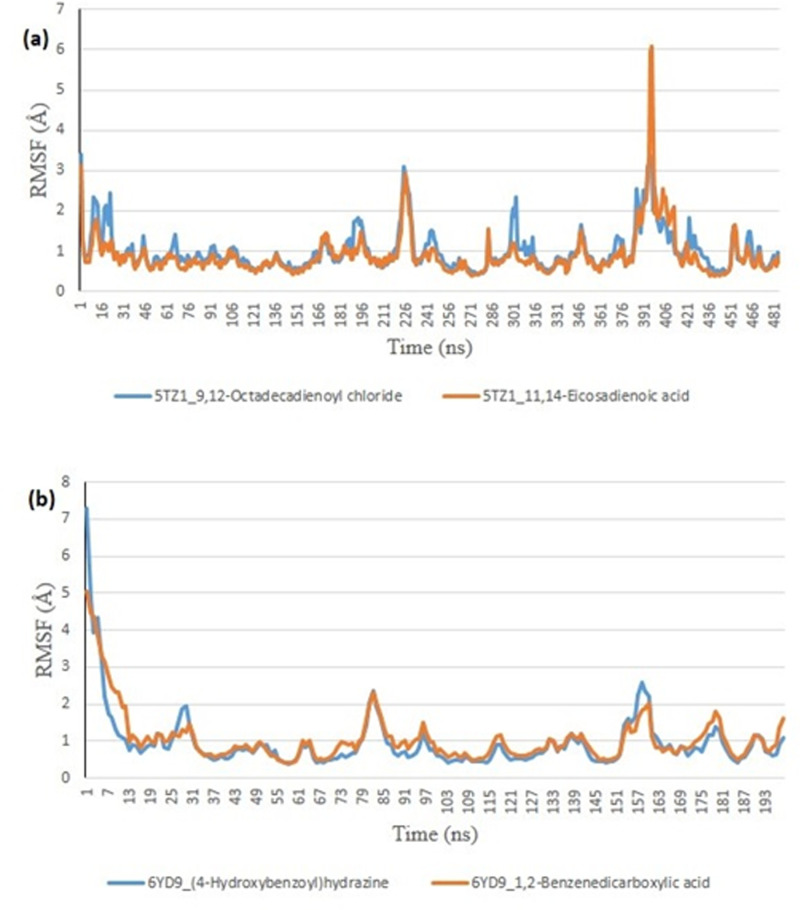
Plots of the root mean square fluctuations (RMSF) per residue for ligands complexed to (a) 5TZ1 and (b) 6YD9 in molecular dynamics (MD) simulation.

**Fig 10:**
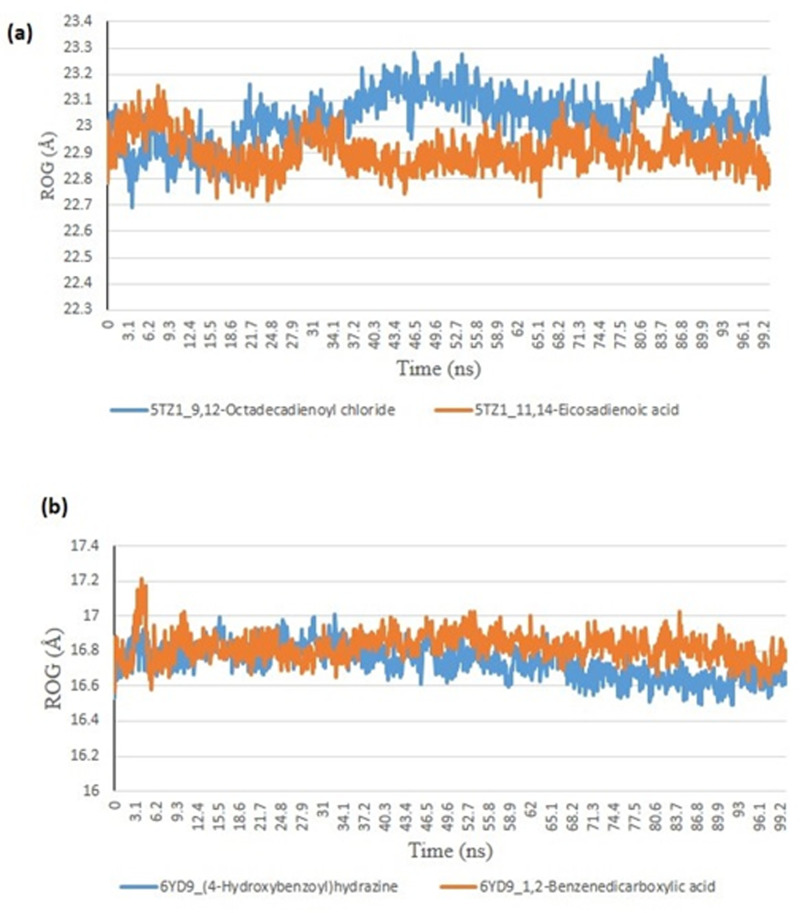
The plots for the radius of gyration (RoG) for molecular dynamics (MD) simulation of ligands complexed to (a) 5TZ1 and (b) 6YD9.

**Fig 11:**
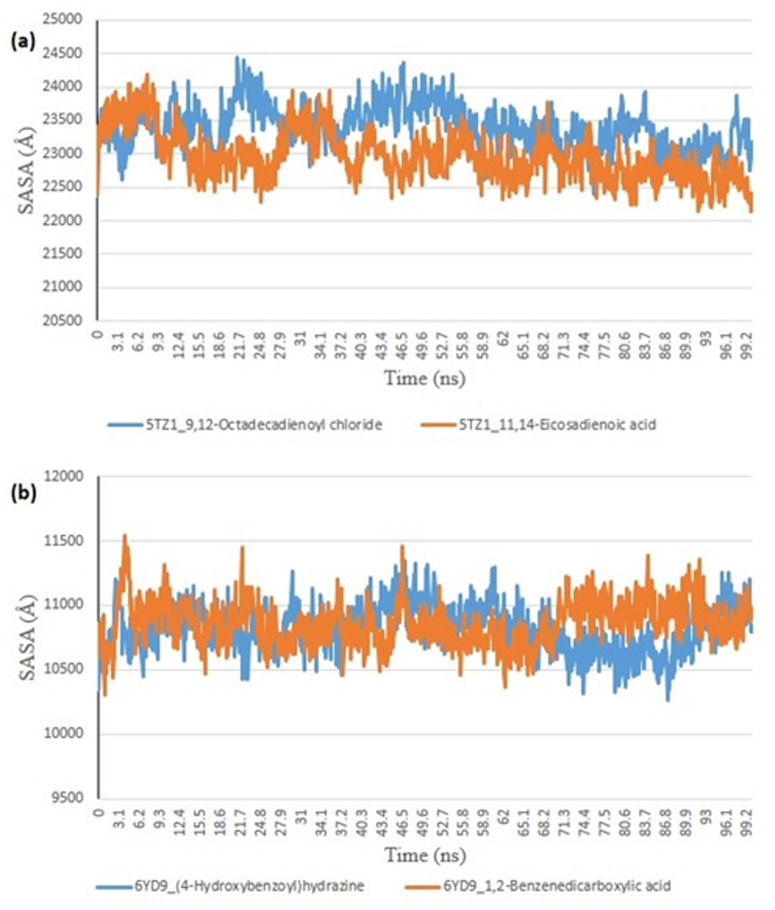
The plots for Surface Accessible Surface Area (SASA) of molecular dynamics (MD) simulation of ligands complexed to (a) 5TZ1 (b) 6YD9.

From the molecular docking study, the stability of the protein complexed with the two top-docked GCMS discovered compounds using the Tk console scripts in VMD version 1.9.3. The number of H-bonds, RoG, SASA, RMSD, and RMSF were used to analyze the trajectories of the simulation (Table 6). Figs 7–11 show the spectra plot for ligand-bond and unattached proteins. The two top-docked compounds complexed with hMOA showed equilibrated RMSD plots that fluctuated little during the simulation, averaging approximately 10 ns (Fig 8). On the other hand, both systems exhibit increased fluctuation after 20 ns, when the RMSD plots for the two top-docked chemicals complexed with hMOA were equilibrated at around 10 ns. The protein-ligand complexes 1xp0_vardenafil, 1xp0_valencene, and 1xp0_alloromadendrene had average RMSD values of 1.4546, 1.4313, and 1.5996 Å, respectively.

**Table 6:** The mean and standard deviation of different parameters analyzed from the MDS trajectories of top-docked compounds complexed with respective targets.

	RMSD	RMSF	RoG	SASA	H-Bonds
	**Mean (Å)**	**Mean (Å)**	**Mean (Å)**	**Mean (Å)**	**Mean (Å)**
5TZ1_9,12-Octadecadienoyl chloride	1.77 ± 0.22	0.99 ± 0.49	23.03 ± 0.09	23403.5 ± 334.08	124.42 ± 11.78
5TZ1_11,14-Eicosadienoic acid	1.77 ± 0.17	0.89 ± 0.55	22.90 ± 0.06	22968.5 ± 370.70	124.5 ± 11.58
6YD9_(4-Hydroxybenzoyl) hydrazine	1.65 ± 0.37	0.95 ± 0.77	16.73 ± 0.09	10814.8 ± 185.4	52.19 ± 11.66
6YD9_1,2-Benzenedicarboxylic acid	1.60 ± 0.23	1.05 ± 0.68	16.82 ± 0.07	10863.4 ± 177.6	51.98 ± 5.64

#### Molecular dynamics.

**Fig 12:**
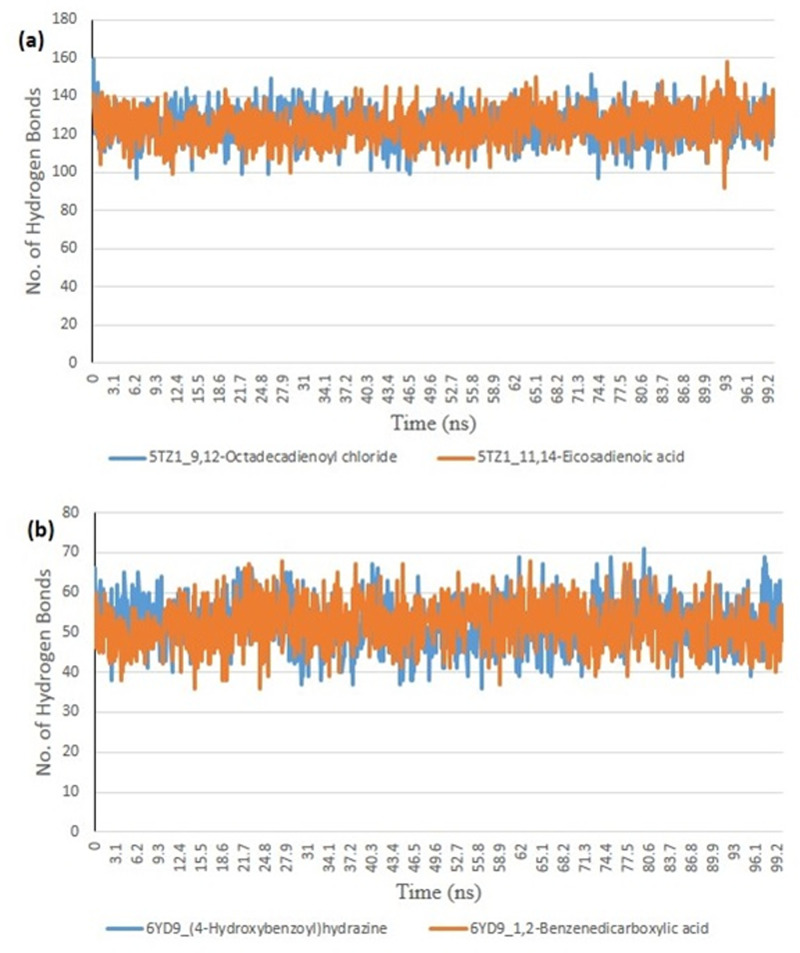
The plots for showing the changes in the number of H-bonds during the MDS trajectory of ligands complexed to (a) 5TZ1 and (b) 6YD9.

During the molecular dynamics, the Tk console scripts in VMD version 1.9.3 were utilized to examine the stability of the complexes formed by the two top-docked phytochemicals with representative proteins. The simulation results for the MD trajectories were used to calculate the RMSD, RMSF, RoG, SASA, and number of H-bonds. All the parameter averages and standard deviations are included in Table 7, and the complex spectrum map is shown in Figs 9–12 All the RMSD plots for the 5TZ1 and 6YD9 complexes were equilibrated before 10 ns, and throughout the remainder of the experiment, neither system encountered any significant equilibration. There was no significant protein distortion following ligand binding, as indicated by the relatively near mean RMSD values for both complexes. A closer RMSF value was shown by the 6YD9, indicating that it was more compacted with the binding of 5TZ1_9,12-Octadecadienoyl chloride, but the RMSF of 5TZ1_11,14-Eicosadienoic acid was lower (Fig 9). Throughout the simulation, the RoG plots demonstrate that both system complexes were equilibrated before 10 ns and saw very little variation (Fig 10). The mean RoG ratings of the top two compounds on the pier were extremely similar. The complexes’ SASA plots demonstrate the little volatility that was seen during the simulation period. The extremely similar mean SASA values further supported this (Fig 11). Throughout the simulation, there were very few variations in the total number of H-bonds. The number of hydrogen bonds in the ligand-bound complexes was near (Fig 12).

**Table 7:** The mean and SD of different energy components that make the binding free energy of top-docked phytochemicals to target proteins.

System	Δ_Vdwaals_	Δ_Eel_	Δ_Egb_	Δ_Esurf_	Δ_Ggas_	Δ_Gsolv_	Δ_Total_
5TZ1_9,12-Octadecadienoyl chloride	−41.39 ± 2.52	−6.67 ± 4.28	29.36 ± 4.39	−6.43 ± 0.36	−48.06 ± 4.86	22.93 ± 4.30	−25.1 ± 2.57
5TZ1_11,14-Eicosadienoic Acid	−44.44 ± 2.85	−5.14 ± 4.79	33.95 ± 3.76	−7.29 ± 0.39	−49.58 ± 5.42	26.67 ± 3.56	−22.91 ± 3.17
6YD9_(4-Hydroxybenzoyl) Hydrazine	−17.40 ± 3.53	−15.92 ± 12.40	25.83 ± 9.17	−2.86 ± 0.48	−33.32 ± 12.59	19.98 ± 8.89	−13.34 ± 4.7
6YD9_1,2-Benzenedicarboxylic Acid	−10.44 ± 6.65	319.73 ± 112.9	−294.9 ± 96.26	−2.28 ± 1.82	−26.5 ± 1.14	16.22 ± 18.05	−12.0 ± 10.61

#### Molecular mechanics generalized born surface area (MMGBSA) analysis.

**Fig 13:**
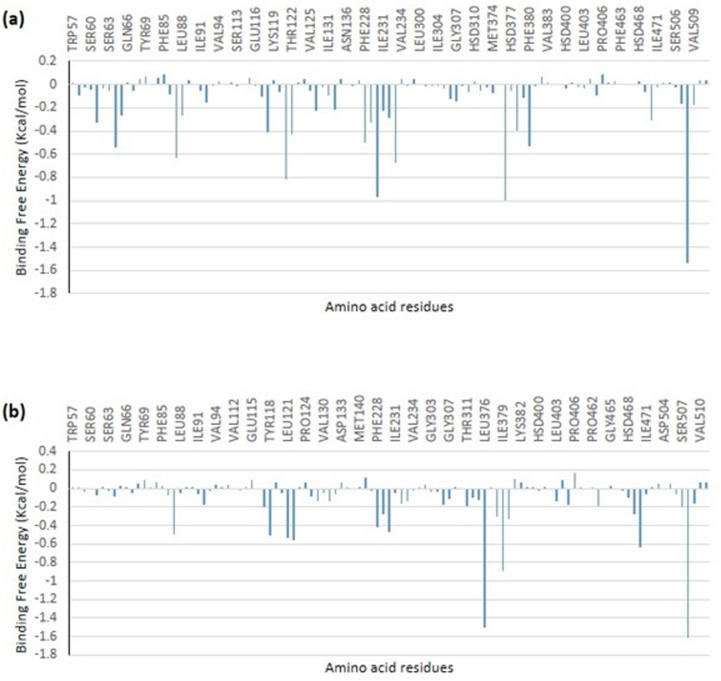
MMPBSA free energy decomposition of residues within 10 Å of 5TZ1 complexed with (a) octadecadienoyl chloride and (b) 11,14-Eicosadienoic acid.

**Fig 14:**
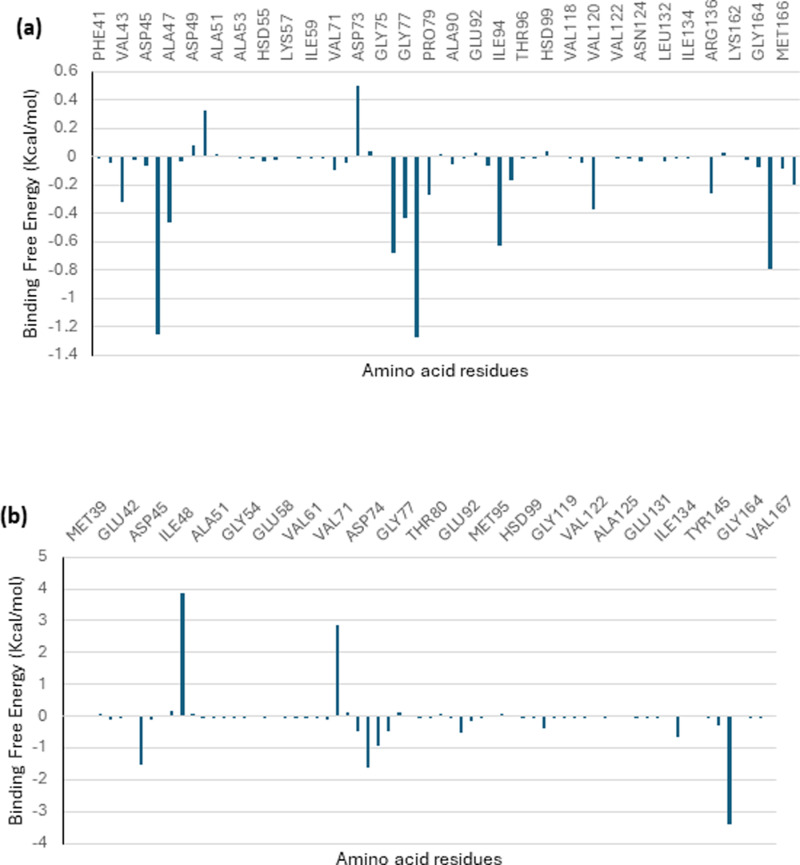
MMPBSA free energy decomposition of residues within 10 Å of 5TZ1 complexed with (a) (4-hydroxybenzoyl) hydrazine (b) 1,2-benzenedicarboxylic acid.

The binding free energy of two of the top-docked phytochemicals to the target proteins was ascertained using the MMGBSA technique. According to the calculated binding free energy, the maximum binding free energy was found for octadecadienoyl chloride with 5TZ1, and the highest binding free energy for (4-hydroxybenzoyl) hydrazine with 6YD9. The binding free energy result was combined with the static binding energy computation’s previous result. Table 7 presents the many components that together comprise the total binding free energy. Using the decomposition technique, the contributing amino acids that were evaluated to determine the overall binding energy are shown in Figs 13 and 14. During static docking, it was discovered that the interacting residues contributed most of the binding free energy.

## Discussion

### Macroscopic and microscopic analysis

The pharmacognostic study aids in identifying the contentious group of plant species and guards against plant adulteration in dry powder [26]. According to Alamgir and Alamgir [48] and Sonibare *et al*. [25], macroscopical evaluation of crude drugs can serve as a diagnostic tool for the accurate and thorough identification of crude drugs and prevent intentional substitution and adulteration with closely related species of crude drugs during the sourcing of medicinal plant. It was easier to identify and standardize *P. aquilinum* leaves because they have fiber, vessels, and epidermal cells but no trichomes. The cellulose cell wall, lignin, oleoresin, tannins, starch granules, lignified elongated fibers and epidermal cells were all found in the powdered leaf of *Pteridium aquilinum* when examined under a microscope. Crystals of calcium oxalate were absent in the plant. Specific color reactions with various reagents enable the identification of the presence of these compounds. Several chemical substances found in the plant exhibit fluorescence [25,49,50]. These features could serve as a benchmark for *P. aquilinum*’s identification and validation.

### Proximate analysis

For determining the quality of crude drugs, physicochemical characteristics including ash levels and moisture content are particularly crucial [27]. Examining the moisture content is crucial because too much water in vegetable medications can promote the growth of bacteria, fungi, and other microorganisms as well as the hydrolysis of compounds, which can damage the raw medication [51]. The moisture content of *P. aquilinum* is within the accepted range for medicinal plants [52,53]. Furthermore, according to Mandal *et al*. [54] and Al-Harrasi *et al*. [53], a lower ash value indicates less contaminants in the plant material.

Plant-based sources of minerals, proteins, lipids, and carbohydrates contribute significantly to the healthy development of the central nervous system in humans [55,56]. Although fiber has minimal or no nutritional value, it is said to help with the management of diabetes and high blood cholesterol due to its impact on digestion and absorption [57]. Amino acids are found in proteins, and these amino acids are utilized by body cells to make all the proteins needed to function normally [58]. Because of its low fat and high fiber contents, *P. aquilinum* leaf might be recommended as a dietary supplement for people with metabolic disorders [27].

### Plant extraction, solvent partitioning, and antioxidant activity

The extraction yield provides insight into the plant’s extractability under various circumstances [27]. Following leaf extraction with methanol, the yield in percentage is 2.06%. Among the four fractions and the crude extract, the ethyl acetate fraction exhibited the best DPPH radical scavenging activity. Numerous biological actions, including anti-inflammatory, anti-microbial, and anti-proliferation properties, are exhibited by antioxidants, which are suspected to be in the DCM fraction [33,59].

### Phytochemistry

Phytochemical screening offers a detailed description of the medicinal potential of the plant [27,30,60,61]. Seven phytochemicals, including saponin, tannins, flavonoids, anthraquinones, alkaloids, steroids, and terpenoids, were identified in the methanol of crude extract from *P. aquilinum* leaves. Anthraquinones, alkaloids, and terpenoids were found in the n-*N-hexane* fraction; anthraquinones, alkaloids, and terpenoids were found in the DCM fraction; and both methanol extract and ethyl acetate fraction contained flavonoids. From quantitative phytochemical quantification, the leaves of *P. aquilinum* were found to possess significantly more flavonoids and less phenol. Flavonoids are biologically active compounds that exhibit anti-microbial, antiviral, anticancer, anti-inflammatory, anti-carcinogenic, anti-aging, and anti-allergenic effects [62,63].

Numerous compounds were identified in the leaf extract, according to the thin-layer chromatography study. Amine, secondary alcohol, ester, aromatic ester, carboxylic acid, conjugated aldehyde, amine salt, alkane, and alcohol were all validated by FTIR analysis. For the accurate identification of chemical constituents in crude pharmaceuticals, gas chromatography-mass spectroscopy (GC-MS) is a useful analytical method [27,64]. A total of eighteen (18) compounds with varying retention times (RTs) were identified in the DCM fraction, including Z-9-Tetradecena ester (24.99%), and 4-Decyl methylphosphonofluoridate (0.88%).

### Antimicrobial activity of *P. aquilinum* leaf

*P. aquilinum* leaf extract and fractions were efficacious against *Candida albicans* and *Aspergillus niger*. Crude extract (CE), ethyl acetate (EAF) fraction and dichloromethane fraction (DCMF) were effective against *S. aureus, S. typhi, E. coli, and P. aeruginosa*. The proliferation of bacteria isolates was consistently inhibited by dichloromethane fraction (DCMF) at all doses. These zones of inhibition for the extract and fractions were like that of the common medication, gentamycin. While the crude extract and other fractions demonstrated considerable microbial activity against the test strains, the dichloromethane fraction showed significantly notable antimicrobial activity. Furthermore, the compounds detected in dichloromethane fraction by GC-MS analysis have been shown to inhibit the growth of both gram-positive and gram-negative bacteria as well as fungi [64–67].

### *In silico* study

Due to their capacity to show the extent of each frame’s departure from the original structure, RMSD plots are used to assess the protein stability of the systems [68]. The RMSF plots show the flexibility of several areas of the enzyme. The lead phytochemicals’ binding did not alter the proteins’ intrinsic flexibility [27]. It was possible to measure the compactness of the bound systems further by analyzing the RoG plots. The solvent accessibility of the protein surface is usually displayed by the SASA plots. Both SAS and RoG are used to examine if ligand interaction affects the folded protein’s integrity [69]. Both analyses indicated that the compactness of the protein structures remained unchanged. Comparatively more accurate and dependable were found to be quantitative simulation-based estimations of the free binding affinity energy of ligands to proteins in a dynamic context [70]. In the early phases of drug design and development, the binding free energy simulations provide thorough information about the binding mechanisms of the best-docked compounds. The breakdown analysis of the total free energy to contributing amino acids and the binding free energy estimate based on the MMGBSA techniques showed that the post-dynamics results confirmed the accuracy of the static docking estimates [27,59,71].

## Conclusion

The bi-pinnate leaflet and alternate pinna arrangement are two of the macroscopic characteristics of *P. aquilinum* leaves. The *P. aquilinum* plant possessed microscopic characteristics such as wavy epidermal cells, vascular bundles, anomocytic stomata, many stomata in the abaxial layer, and an absence of stomata in the adaxial layer. The *P. aquilinum* leaf dichloromethane fraction showed a strong antimicrobial effect, and using *in silico* studies, 4-hydroxybenzoyl) hydrazine, 1,2-benzenedicarboxylic acid, octadecadienoyl chloride, and 11,14-Eicosadienoic acid, identified in the DCM fraction of *P. aquilinum* leaves, offered the highest binding free energy to 5TZ1 and 6YD9. Thus, *P. aquilinum* leaf possessed significant antioxidant and antidiabetic activities. Therefore, bioassay-guided isolation is hereby recommended.

## Supporting information

S1 File Supplementary data. (DOCX)
