# Supplementary material for: Pharmacognostic evaluation and antimicrobial activity of Pteridium aquilinum (L.) Kuhn leaves (Onocleaceae) via in vitro and in silico perspectives
Source: PLoS One. 2025 Apr 9;20(4):e0318943. doi: 10.1371/journal.pone.0318943 (PMC11981126; doi:10.1371/journal.pone.0318943)
Supplement: S1 File — (DOCX) [file pone.0318943.s001.docx]

**Pharmacognostic Evaluation and Antimicrobial Activity of *Pteridium aquilinum* (L.) Kuhn Leaves (Onocleaceae): *in vitro* and *in silico* perspectives**

Oluwatoyin Temilolu Adebayo ^a^, Bolaji Bosede Oluremi ^b^, Akingbolabo Daniel Ogunlakin ^c,*^, Oluwafemi Adeleke Ojo ^c^, Gideon Ampoma Gyebi ^d^, Mubo Adeola Sonibare ^a^

**Supplementary data**


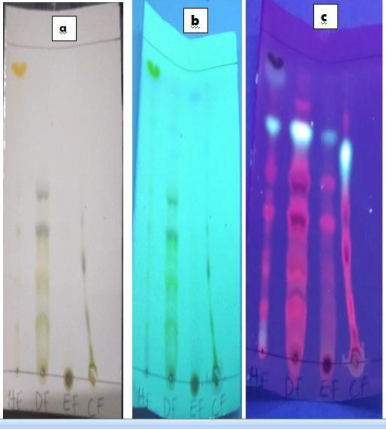


**Figure S1**: TLC plate of the crude extract and fractions of *P. aquilinum* leaf

Solvent system: Ethyl acetate 1: Hexane: 4. Visualization: **a:** Daylight, **b**: 254 nm, **c**: 365 nm.


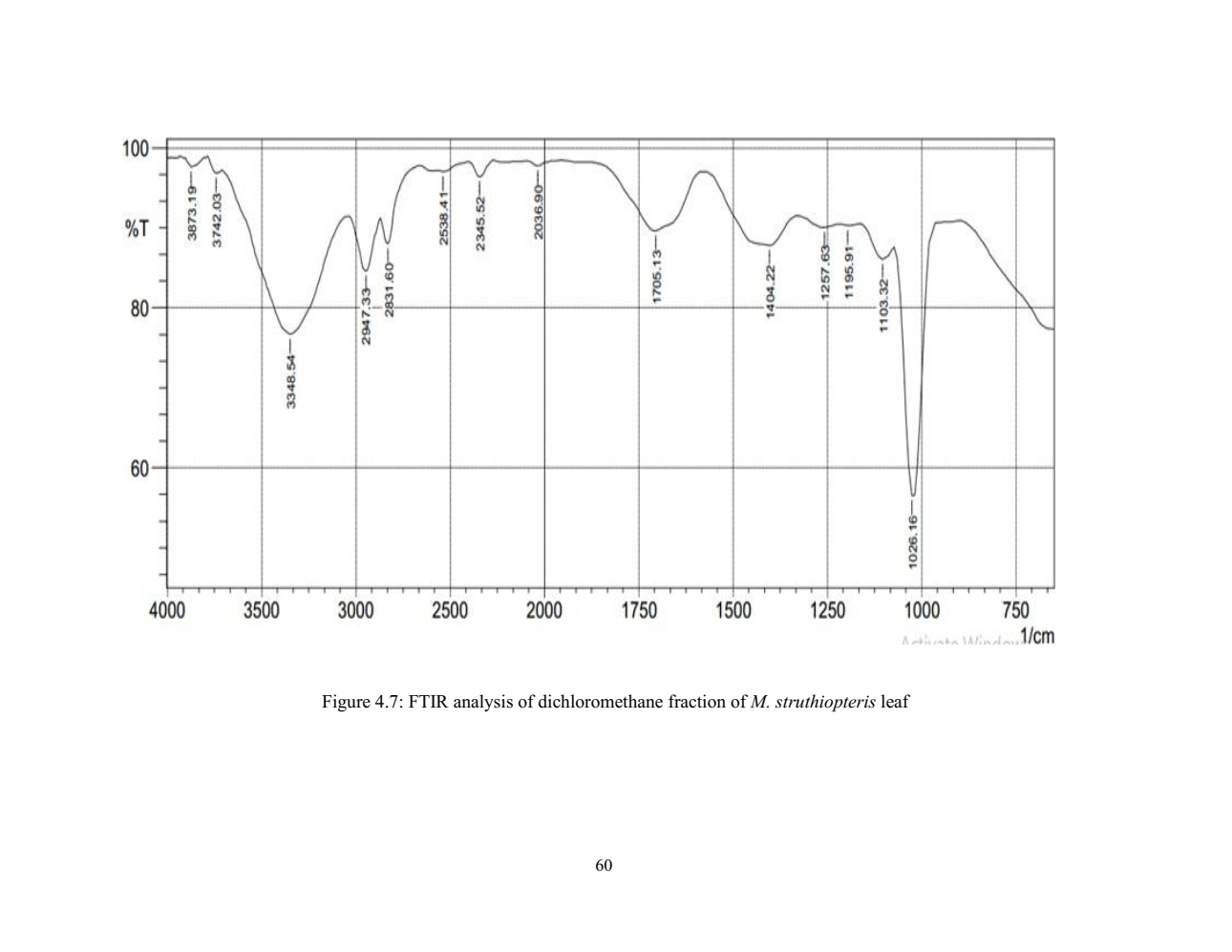


**Figure S2**: FTIR analysis of dichloromethane fraction of P. aquilinum *l*eaf

**Table S1:** Target protein binding coordinates

| **Dimensions** | **5TZ1 (Å)** | **6YD9 (Å)** |
| --- | --- | --- |
| center_x | 70.27 | 10.67 |
| center_y | 65.79 | -4.79 |
| center_z | 3.64 | 6.95 |
| Size x | 3.64 | 6.95 |
| Size y | 22.60 | 18.70 |
| Size z | 22.72 | 18.70 |

**Table S2: Macroscopy of *P. aquilinum***

| **Character** | **Observation** |
| --- | --- |
| Leaf apex | Ternate |
| Leaf base | Truncate |
| Leaf margin | Entire |
| Leaf shape | Oblique |
| Leaf colour | Light green |
| Leaf texture | Smooth |
| Leaf arrangement on the stem | Alternate |
| Venation pattern | Furcate |
| Leaf length (cm) | 0.3-2.6 |
| Taste | Tasteless |
| Odour | Odourless |
| **Powder study test** | |
| Colour | Brown |
| Odour | Odourless |
| Taste | Tasteless |

**Table S3:** Histology of *P. aquilinum* leaf

| **Parameters** | | A**baxial** | **Adaxial** |
| --- | --- | --- | --- |
| 1. | Stomata | Present | Absent |
| 2. | Stomata type | Anomocytic | Absent |
| 3. | Epidermal shape | Wavy | Wavy |
| 4. | Trichome | Absent | Absent |

**Table S4:** Microscopy of the powdered sample of *Pteridium aquilinum* leaf

| **Parameters** | **Observation** |
| --- | --- |
| Epidermal cell | + |
| Fibre | + |
| Vessels | + |
| Trichome | - |

**Table S5:** Fluorescence analysis of *P. aquilinum* powdered leaf

| **Extract** | **Daylight** | **254 nm** | **365 nm** |
| --- | --- | --- | --- |
| Picric acid | yellow | Light green | purple |
| FeCl_3_ | Brown | Light green | purple |
| Acetic acid | Brown | Light green | Yellow |
| 50% HCl | Brown | Light green | Purple |
| 50% H_2_SO_4_ | Light green | Green | Purple |
| Methanol | Light green | Green | Red |
| Ethanol | Light green | Green | Light green |
| Water | Colourless | Colourless | Light green |

**Table S6:** chemo-microscopic character of dried powder leaf of *P. aquilinum*

| **Reagent** | **Observation** | **Inference** |
| --- | --- | --- |
| N50 iodine | Blue-black color | Starch present |
| Phloroglucinol + conc. HCl | Red stain | Lignin present |
| Sudan red and | Pinkish red colour | Fibre present |
| Rheutenium red | Red | Mucilage present |
| HCl | No crystals | Calcium oxalate absent |

**Table S7:** Proximate parameters of *P. aquilinum* powdered leaf

| **Physiochemical parameter** | **Composition (%)** |
| --- | --- |
| Moisture content | 11.15±0.50 |
| Crude carbohydrates | 33.04±0.02 |
| Crude fat | 0.44±0.24 |
| Crude protein | 0.83±0.03 |
| Crude fiber | 45.64±0.21 |
| Total ash content | 4.7±0.00 |

**Table S8:** Percentage yield of methanol extract of *P. aquilinum* leaf

| **Extract** | **Weight of dry sample (g)** | **Weight of extract (g)** | **% yield** |
| --- | --- | --- | --- |
| Crude | 900 | 18.54 | 2.06 |

**Table S9:** Qualitative phytochemical screening of *P. aquilinum* leaf

| **Test** | **n-hexane** | **DCM** | **Ethyl acetate** | **Methanol** | **Crude** |
| --- | --- | --- | --- | --- | --- |
| Saponin | - | - | + | ++ | ++ |
| Tannins | - | - | - | + | + |
| Flavonoids | - | - | ++ | + | + |
| Cardiac glycosides | - | - | + | + | + |
| Anthraquinones | ++ | + |  | + | + |
| Alkaloids | + | + | ++ | + | + |
| Steroids | - | - | - | + | + |
| Terpenoids | ++ | + | ++ | ++ | + |

**Table S10:** Fourier transform infrared spectroscopy analysis of dichloromethane fraction of *P. aquilinum* leaf

| **S/N** | **Wave number (cm^-1^)** | **Intensity** | **Area** | **Functional groups** | **Phytocompounds Identified** |
| --- | --- | --- | --- | --- | --- |
|  | 1026.16 | 56.552 | 17.321 | C-N stretching | Amine |
|  | 1103.32 | 86.101 | 5.205 | C-O stretching | Secondary alcohol |
|  | 1195.91 | 90.285 | 2.371 | C-O stretching | Ester |
|  | 1257.63 | 90.073 | 4.645 | C-O stretching | Aromatic ester |
|  | 1404.22 | 87.816 | 10.312 | O-H bending | Carboxylic acid |
|  | 1705.13 | 89.625 | 8.312 | C=O stretching | Conjugated aldehyde |
|  | 2036.9 | 97.769 | 1.078 | N=C=S stretching | Isothiocyanate |
|  | 2345.52 | 96.403 | 1.536 | O=C=O stretching | Carbon dioxide |
|  | 2538.41 | 97.056 | 1.602 | O-H stretching | Carboxylic acid |
|  | 2831.6 | 88.006 | 5.723 | N-H stretching | Amine salt |
|  | 2947.33 | 84.628 | 9.392 | C-H stretching | Alkane |
|  | 3348.54 | 76.715 | 46.591 | O-H stretching | Alcohol |
|  | 3742.03 | 96.899 | 0.858 | O-H stretching | Alcohol |
|  | 3873.19 | 97.657 | 0.826 | O-H stretching | Alcohol |

**Table S11:** Anti-fungal activity of crude extract and fractions of *P. aquilinum*

|  | **Zones of inhibition produced by different antibiotics (mm)** | | | | | | | | | | | | | | | |
| --- | --- | --- | --- | --- | --- | --- | --- | --- | --- | --- | --- | --- | --- | --- | --- | --- |
| **Isolates** | **CE** | | | **EEF** | | | **HF** | | | **DCMF** | | | **AQF** | | | **FC** |
|  | **25** | **50** | **100** | **25** | **50** | **100** | **25** | **50** | **100** | **25** | **50** | **100** | **25** | **50** | **100** | **50** |
| *C. albicans* | 18 | 16 | 14 | 12 | 16 | 18 | 18 | 10 | 12 | 14 | 16 | 18 | 12 | 0 | 0 | 10 |
| *A. niger* | 24 | 18 | 10 | 12 | 18 | 20 | 0 | 10 | 12 | 12 | 18 | 24 | 12 | 10 | 12 | 10 |

Key: CE: Crude extract, EAF: Ethyl acetate fraction, HF: *N-hexane* fraction, DCMF: Dichloromethane fraction, AQE: Aqueous fraction, FC: Fungi control (ketoconazole).

**Table S12:** Anti-bacterial activity of crude extract and fractions of *P. aquilinum*

| **Zones of inhibition produced by different antibiotics (mm)** | | | | | | | | | | | | | | | | |
| --- | --- | --- | --- | --- | --- | --- | --- | --- | --- | --- | --- | --- | --- | --- | --- | --- |
| **Isolates** | **CE** | | | **EAF** | | | **HF** | | | **DCMF** | | | **AQE** | | | **BC** |
|  | **25** | **50** | **100** | **25** | **50** | **100** | **25** | **50** | **100** | **25** | **50** | **100** | **25** | **50** | **100** |  |
| *S. aureus* | 12 | 16 | 18 | 10 | 10 | 20 | 0 | 8 | 10 | 14 | 16 | 22 | 0 | 0 | 0 | 22 |
| *S. typhimurium* | 10 | 12 | 14 | 8 | 12 | 14 | 0 | 0 | 8 | 12 | 14 | 14 | 14 | 16 | 18 | 18 |
| *E. coli* | 10 | 12 | 14 | 10 | 10 | 16 | 0 | 0 | 10 | 10 | 12 | 14 | 0 | 8 | 12 | 18 |
| *P. aeruginosa* | 14 | 12 | 14 | 12 | 12 | 16 | 0 | 0 | 10 | 14 | 16 | 18 | 0 | 8 | 10 | 14 |

Key: CE: Crude extract, EAF: Ethyl acetate fraction, HF: *N-hexane* fraction, DCMF: Dichloromethane fraction, AQE: Aqueous fraction, BC: Bacterial control (gentamycin).


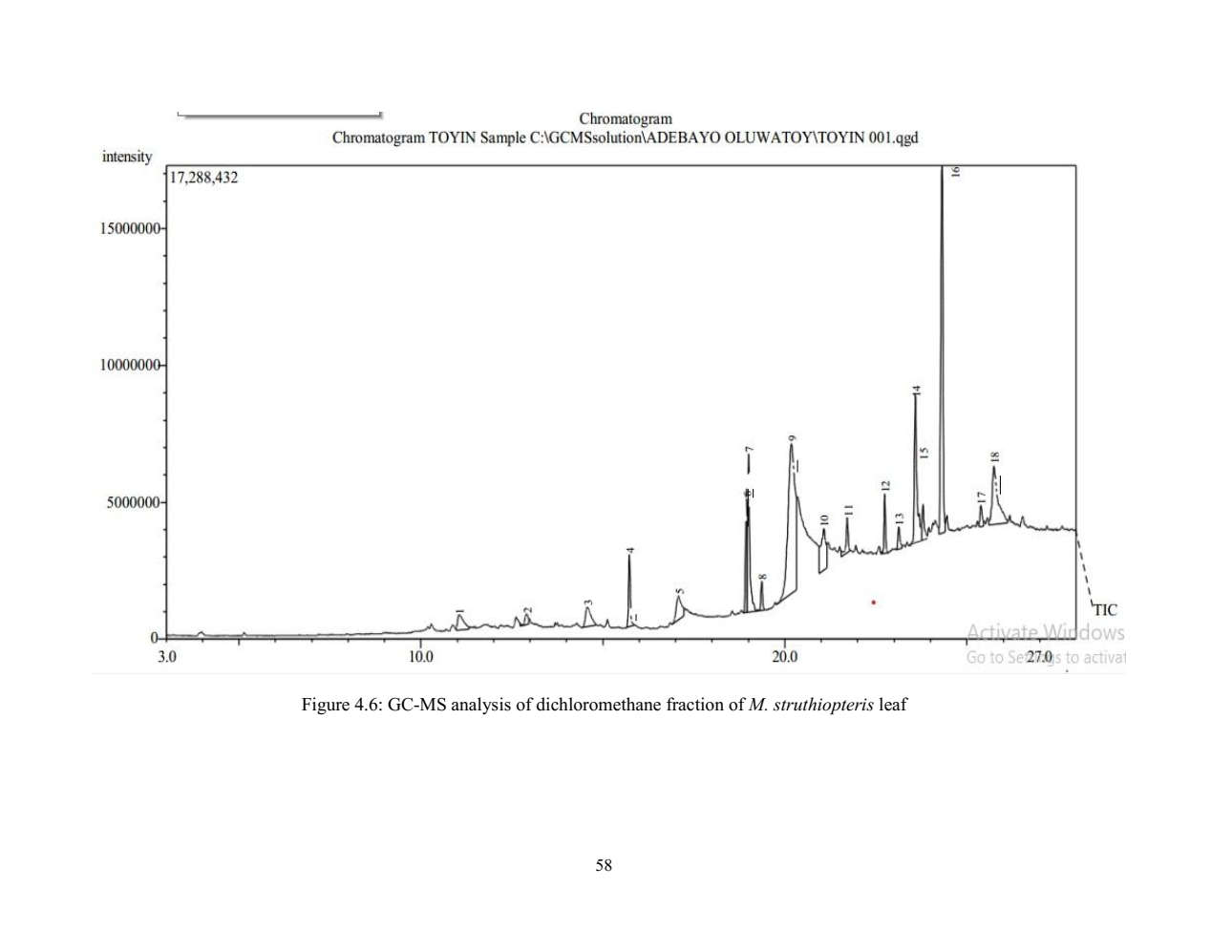


Figure S3: GC-MS analysis of dichloromethane fraction of *P. aquilinum* leaf.
